# Supplementary material for: Modular stimuli-responsive hydrogel sealants for early gastrointestinal leak detection and containment
Source: Nat Commun. 2022 Nov 27;13:7311. doi: 10.1038/s41467-022-34272-y (PMC9701692; doi:10.1038/s41467-022-34272-y)
Supplement: Supplementary file 1 — Supplementary Information [file 41467_2022_34272_MOESM1_ESM.pdf]

Supplementary Material

Modular stimuli-responsive hydrogel sealants for early gastrointestinal leak detection and containment

*Alexandre H.C. Anthis,[1,2] Maria Paulene Abundo,[3] Anna L. Neuer,[1,2] Elena Tsolaki, [1,2] Jachym Rosendorf, [4,5] Thomas Rduch, [2,6] Fabian H.L. Starsich,[1,2] Bernhard Weisse,[7] Vaclav Liska, [4,5] Andrea A. Schlegel,[8,9,10] Mikhail G. Shapiro[3] and Inge K. Herrmann[1,2]**

[1]Nanoparticle Systems Engineering Laboratory, Department of Mechanical and Process Engineering, ETH Zurich, Sonneggstrasse 3, CH-8092 Zurich, Switzerland.

[2]Laboratory for Particles Biology Interactions, Department Materials Meet Life, Swiss Federal Laboratories for Materials Science and Technology (Empa), Lerchenfeldstrasse 5, CH-9014, St. Gallen, Switzerland.

[3]Division of Chemistry and Chemical Engineering, California Institute of Technology, Pasadena, CA, 91125, USA.

[4]Department of Surgery, Faculty of Medicine in Pilsen, Charles University, Prague, Czech Republic

[5]Biomedical Center, Faculty of Medicine in Pilsen, Charles University, Prague, Czech Republic.

[6]Department of Gynaecology, Cantonal Hospital St Gallen (KSSG), Rorschacherstrasse 95, CH-9007, St Gallen, Switzerland.

[7]Laboratory for Mechanical Systems Engineering, Department of Engineering Sciences, Empa - Swiss Laboratories for Materials Science and Technology, Ueberlandstrasse 129, CH-8600 Dübendorf, Switzerland.

[8]Department of Visceral Surgery and Transplantation, University Hospital Zurich

[9]Swiss HPB and Transplant Center, Zurich, Rämistrasse 100, 8091 Zurich, Switzerland.

[10]Fondazione IRCCS Ca' Granda, Ospedale Maggiore Policlinico, Centre of Preclinical Research, Milan 20122, Italy.

Correspondence should be addressed to:

*ingeh@ethz.ch, +41 (0)58 765 7153

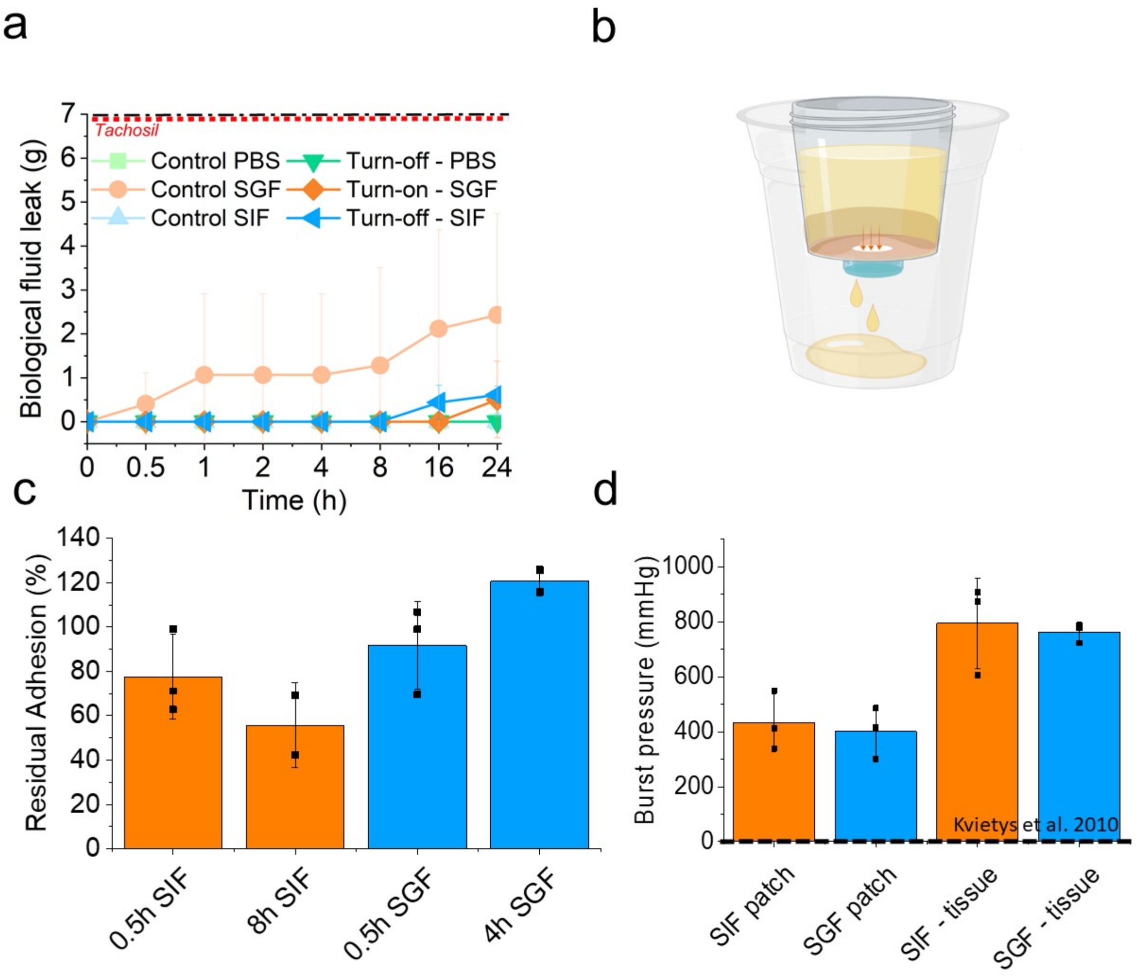

Figure S1: Intestinal fluid leakage model. *(a) Ex-vivo stationary model simulating the pooling of intestinal fluid and evaluating the leak containment imparted by the sealing of a 4 mm open hole in the tissue, covered by the sealant patch. Values for Tachosil included for reference. Leaked fluid mass of PBS, SIF and SGF, respectively, are monitored over time (up to 24 hours). Controls indicate tissue without hole. n = 3 independent cup experiments per condition. Data shown as mean ± standard deviation. (b) Illustration of the ex-vivo stationary model setup. (c) Residual adhesion of applied patches as a function of contact time with digestive fluids (full immersion) at 37 °C and shaking. n = 3 (independent experiments). Data shown as mean ± standard deviation. (d) Burst pressure of adhesive patches under conditions of contact with digestive effluents SIF and SGF (dotted line denotes the maximum native intestinal pressure). n = 3 (independent experiments). Data shown as mean ± standard deviation. Figure wide colour coding: orange – Simulated intestinal fluid conditions, blue – Simulated gastric fluid conditions. Figure 1b has been created using biorender.com.*

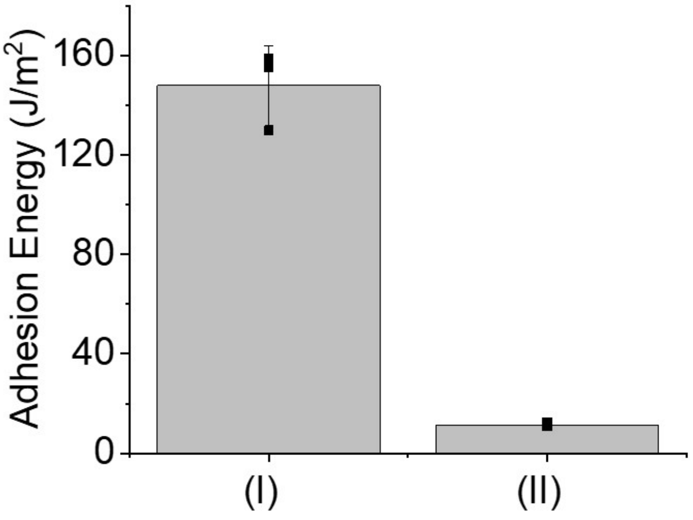

Figure S2: Adhesion energy with or without mIPN. *Adhesion energy (J/m^2) as a function of sample type in the T-peel setup of layered patches after application to porcine small intestine. (I) Layered patches consisting of a PAMPS support layer, a PNHEA backing, applied to tissue using a mutually interpenetrating network of PNAGA joining tissue and patch. (II) Layered patches consisting of a PAMPS support layer, a PNHEA backing and an interpenetrating network of PNAGA applied to tissue. n = 3 (independent experiments). Data shown as mean ± standard deviation. Figure wide colour coding: grey – adhesion energy.*

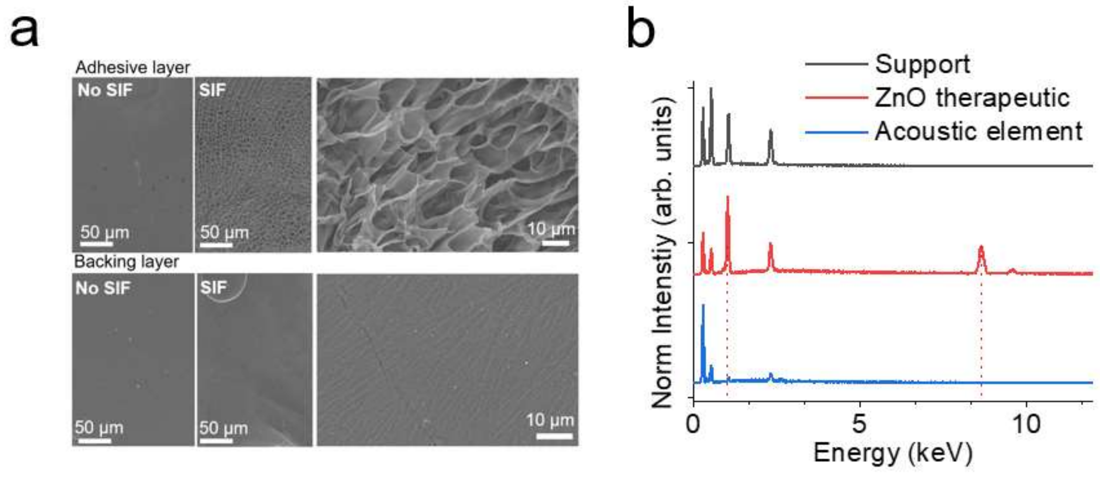

Figure S3: Scanning electron micrographs of adhesive and backing layer before and after SIF exposure. *(a) SEM images of adhesive and backing layer following on sided contact with SIF, simulating a one sided contact with digestive fluid. Samples were analysed in duplicate. (b) EDX elemental analysis of side-cut comprising ZnO therapeutic element, PAMPS support layer and Halo gas vesicle TurnOFF element. Red dotted lines denote the ZnLα and ZnKα peak locations of zinc.*

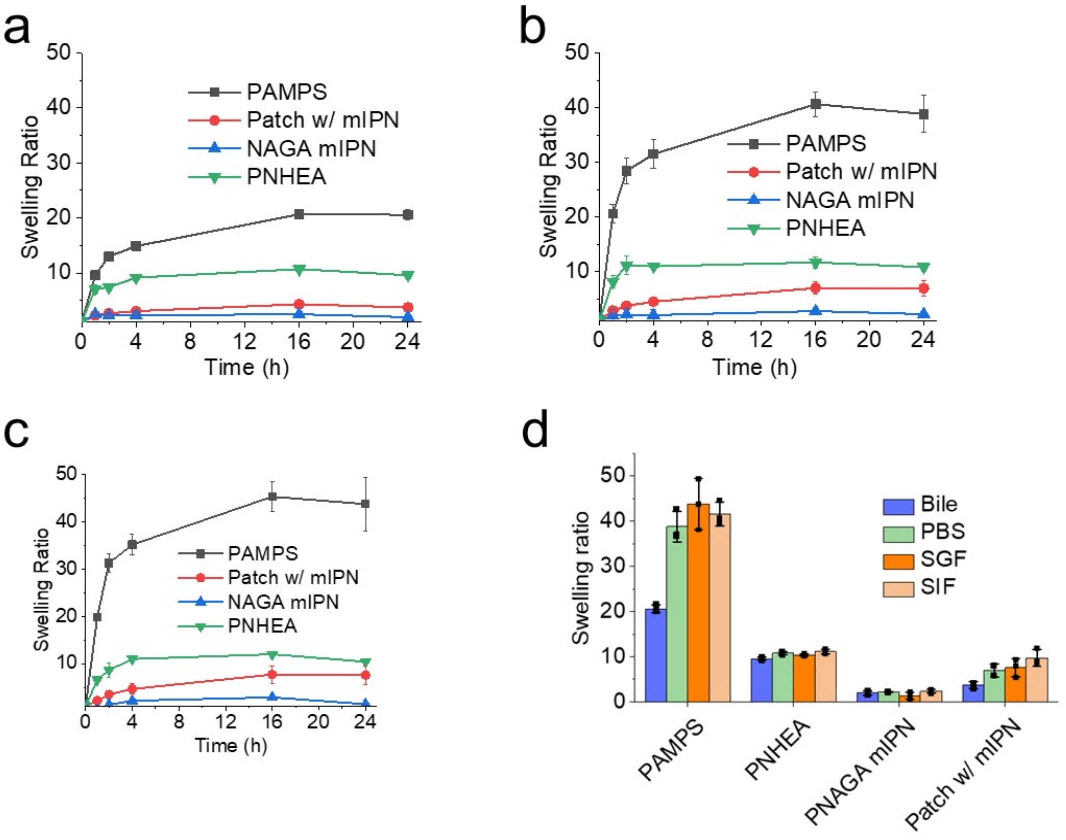

Figure S4: Swelling behavior as a function of time and biological fluid. *Swelling of individual layers as well as fully assembled hydrogel patch after as a function of time in various simulated biological fluids at 37 °C.(a) Simulated gastric fluid, (b) Bile, (c)PBS, (d) 24 h swelling plateaus in different biological fluids. n = 3 (independent experiments). Data shown as mean ± standard deviation. Figure wide colour coding: black – PAMPS, red – Patch with mIPN, blue – NAGA mIPN, green – PNHEA, light blue – Bile, light green – PBS, orange – SGF, light orange - SIF.*

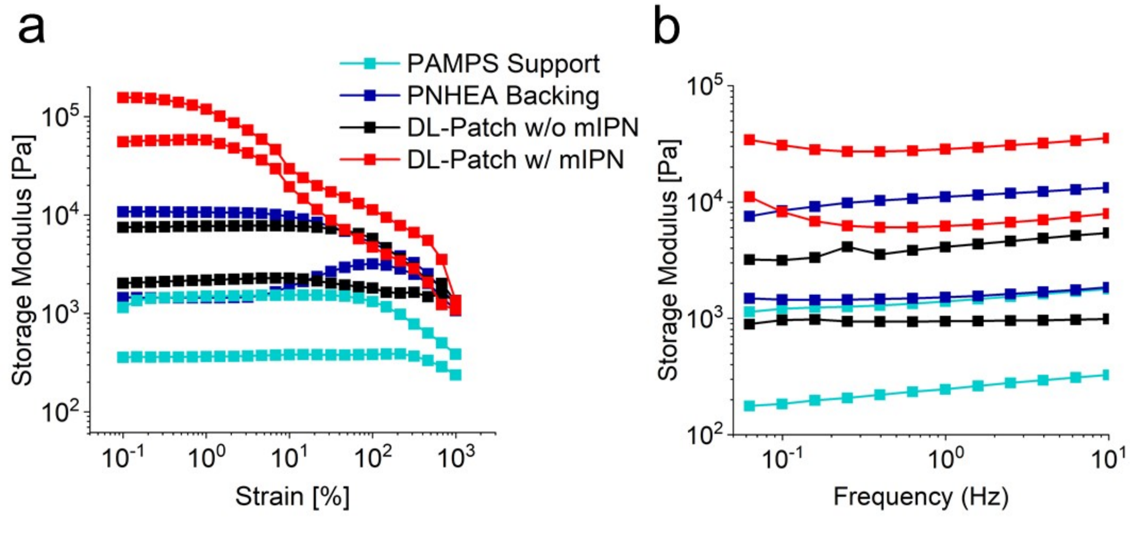

Figure S5**:** ***Rheological properties of hydrogel components and fully assembled DL-Patches.*** *Storage modulus is shown as function of (a) strain and (b) frequency. Figure wide colour coding: light blue – PAMPS support, red – Patch with mIPN, deep blue – PNHEA backing, black – Patch without mIPN.*

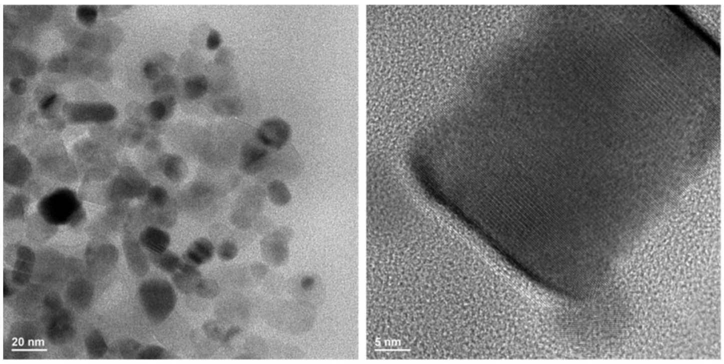

Properties of ZnO NPs	
BET	30 m²/g
XRD	Crystallinity: wurzite<br>d_{XRD}: 21nm
TEM	d_{TEM}: 24 nm (σ_g: 1.4)

Figure S6: ZnO nanoparticles characterization used as a model inorganic antimicrobial. *Transmission electron microscopy (TEM) images depicting ZnO nanoparticles (top left, scale bar: 20 nm) and high resolution TEM images of selected ZnO nanoparticle (top right, scale bar 5 nm). Table summarizing the surface area, crystal structure and size of used ZnO nanoparticles. TEM samples were collected from two independently synthesized ZnO batches yielding data well in line with each other.*

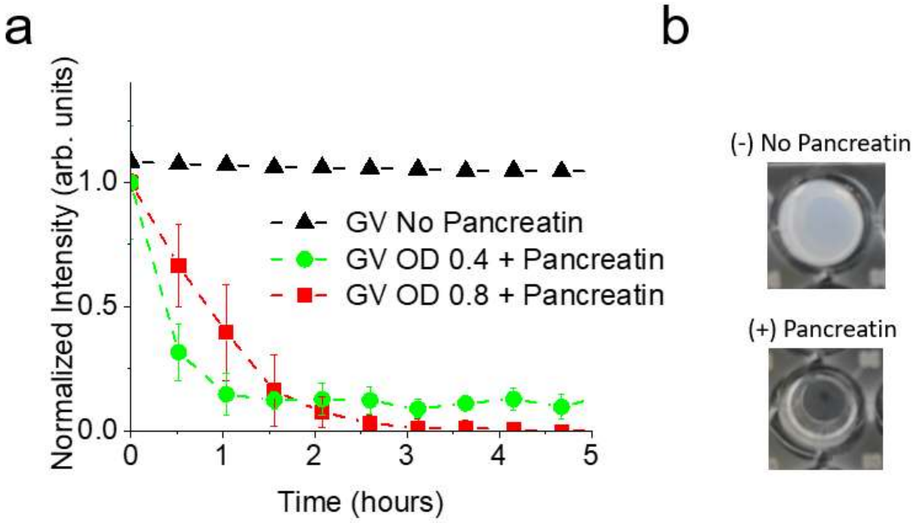

Figure S7: Gas vesicle digestion kinetics. *(a) Digestion kinetics of gas vesicles following incubation with porcine pancreatin enzyme. OD 0.4 corresponding to 20% vol solution in SIF salt background. (b) OD 0.8 in absence and presence of digestive enzyme. n = 3 (independent samples). Data shown as mean ± standard deviation. Figure wide colour coding: green – GV of OD 0.4 with pancreatin, red – GV of OD 0.8 with pancreatin, black – GV without pancreatin.*

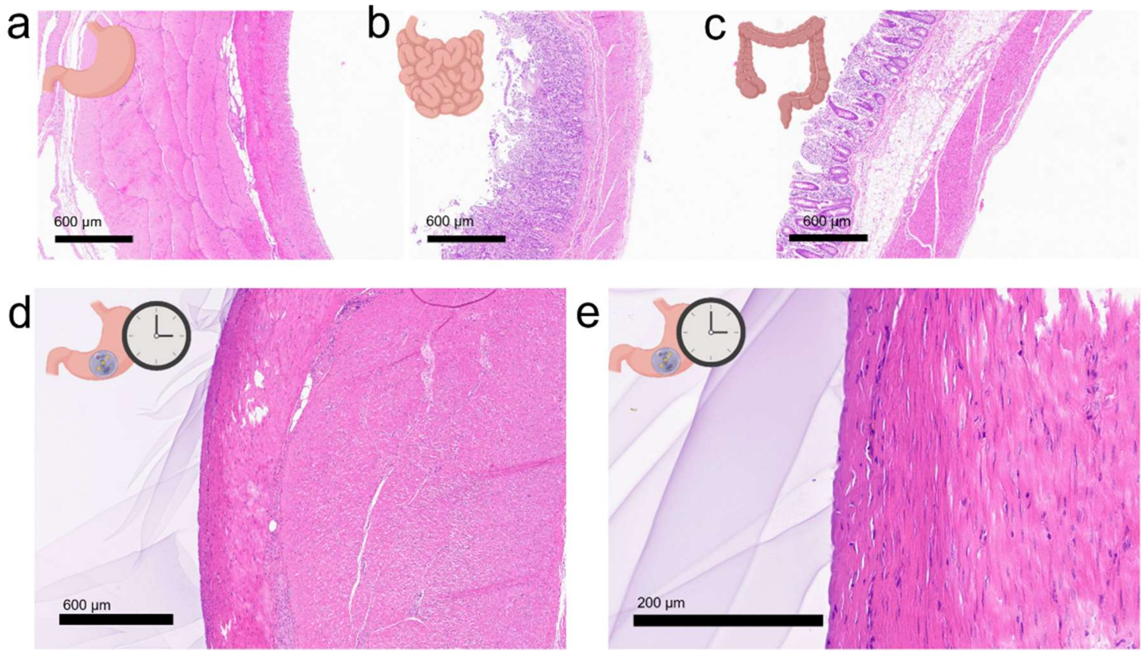

Figure S8: Gastrointestinal tissue biopsies. *Attachment and interfacing of hydrogel sealant on various porcine tissues as showcased by H&E stained biopsy sections. (a –c) Bare tissue control samples showcasing native tissue serosa, (d-e) Representative images of tissue serosa with applied patch after 2h incubation with SGF. Histological samples were prepared from duplicates yielding results well in line with each other. Figure S8a-e have been created using biorender.com.*

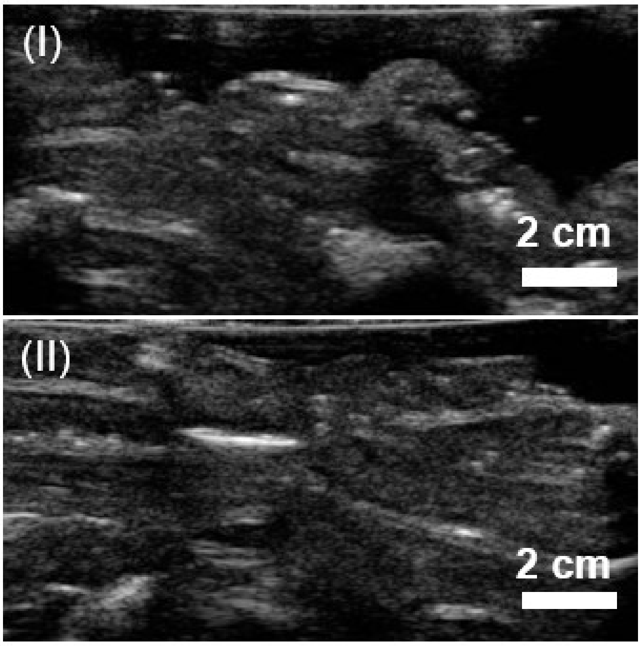

Figure S9: Ultrasound imaging of DL-Patches on intestine. *Identification of gas vesicle loaded sensing element patch in an abdomen simulating model. (I) Native non-sealed porcine small intestine. (II) TurnOFF sealant patch attached on small porcine intestine.*

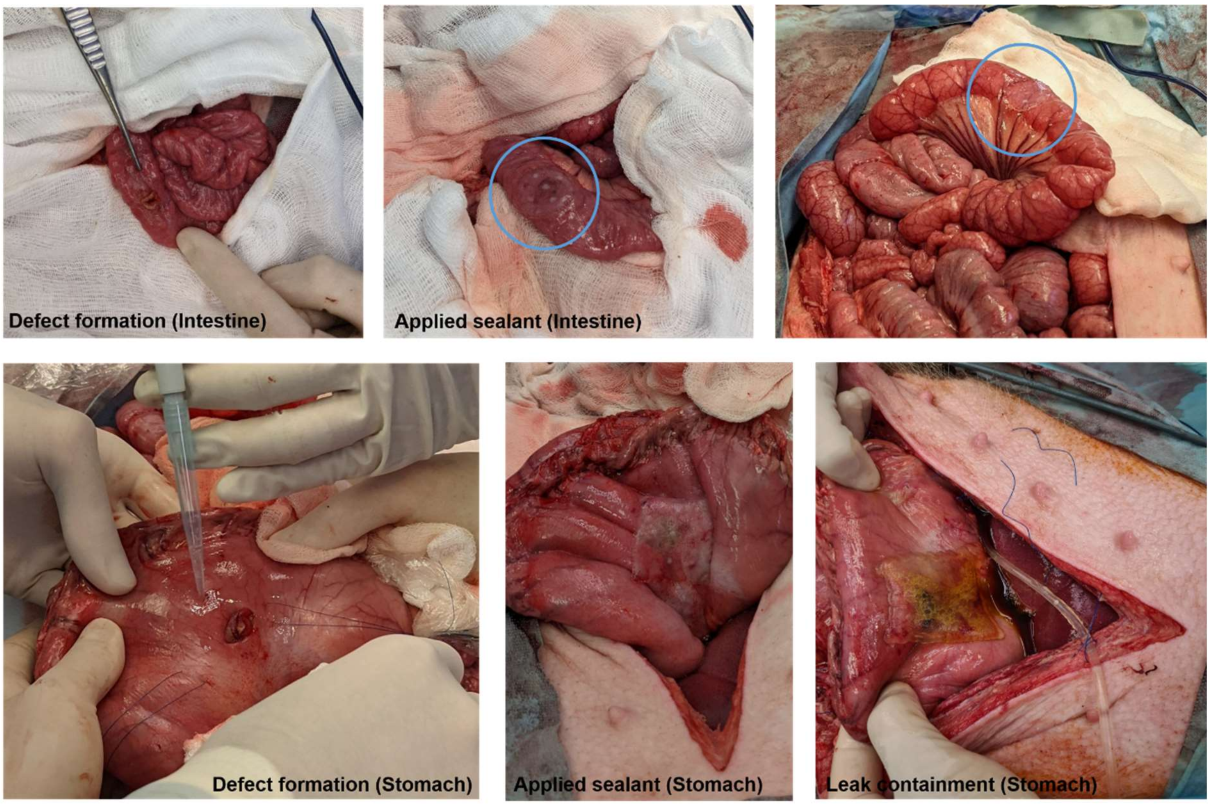

Figure S10: Porcine model of gastrointestinal leaking. *Defect formation at the level of the small intestine and stomach, followed by DL-Patch application. Effective leak containment and firm patch adhesion even in presence of direct contact with SGF following a large defect (1x1 cm hole) on the stomach.*
